# Residential care staff are the key to quality of health care for adults with profound intellectual and multiple disabilities in Sweden

**DOI:** 10.1186/s12913-022-07641-y

**Published:** 2022-02-19

**Authors:** Marie Matérne, Marie Holmefur

**Affiliations:** 1 Faculty of Medicine and Health, University Health Care Research Center, Örebro University, Örebro, Sweden; 2 School of Law, Psychology and Social Work, Örebro University, Örebro, Sweden; 3 School of Health Sciences, Faculty of Medicine and Health, Örebro University, Örebro, Sweden

**Keywords:** Health care, Content analysis, Profound intellectual and multiple disabilities (PIMD), Social care, Quality of care

## Abstract

**Background:**

People with profound intellectual and multiple disabilities (PIMD) have combined severe intellectual and physical disability and need extensive health care support. They cannot communicate by spoken language and need around the clock support. The health care for people with PIMD is typically provided by a number of different health care services in collaboration with residential care staff and their managers. The quality of health care for people with PIMD are important due to their limited ability to communicate their needs. The aim of this study was to explore residential care staff and manager’s experiences and views of health care services for adults with PIMD.

**Methods:**

Thirteen semi-structured interviews with residential care staff (*n* = 7) and managers (*n* = 6) were conducted and analysed using qualitative content analysis.

**Results:**

The informants expressed a variety of experiences, under the theme was *Quality of health care is enhanced through residential care staff.* The theme was comprised of four subthemes: (1) Individually tailored support promotes quality, (2) Accessibility requires adaptation and prioritization by healthcare providers, (3) Disability competence promotes quality and safety and (4) Complex collaboration conditions between the person with PIMD, residential care staff and disability health care.

**Conclusions:**

The residential care staff create quality of care in their role as representatives for adults with PIMD. The care situation is complex and requires adequate competence in the disability, the individual’s needs and adaptations to ensure quality of health care. It is also important to build collaboration with other services that are involved in the care of people with PIMD.

## Introduction

People with profound intellectual and multiple disabilities (PIMD) have a combination of profound intellectual disability (IQ < 20) and inability to move independently [1–3]. Commonly, these people also have sensory impairments and major health problems such as epilepsy and spasticity [2–5]. Causes of PIMD include chromosomal abnormalities, brain malformations, oxygen deficiency or fetal infection and acquired postnatal damage through early onset and progressive brain disease or severe accident [3]. The latest estimate for the prevalence of PIMD in Sweden, was about 80 per 100,000 inhabitants [6]. As a consequence of their severe disability, people with PIMD have limited communication capability, which makes it difficult to convey feelings, thoughts and needs [2].

Adults with PIMD in Sweden are typically financially supported by the Swedish Social Insurance Agency through sickness compensation, and they have daily activity at day centres within the municipality. Further, they typically live in residential care home or in their own homes with staff available around the clock [3]. Their support needs are specific and complex, demanding special care, due to their limited self-care, communication abilities and additional health issues [4, 7]. To fulfil their health care needs, several different care services are involved such as highly specialized care, primary health care and habilitation services, involving many different professionals and others like their next of kin in a sometimes-complicated network. Their everyday health care, is provided by the residential care staff (hereafter mentioned as care staff), typically after instruction from health professionals.

Earlier research focusing on support for people with PIMD has provided evidence for specific interventions enhancing quality of life and care [8, 9]. Other studies have shown that the organization in terms of staff turnover, time spent in active support etc. and characteristics of care staff, such as experience, sensitive responsiveness and physical strength have an important influence on the quality of life of people with PIMD [10, 11]. However, more knowledge is needed on how quality of care is ensured for people with PIMD who have specific and often-complex health care needs. Quality of health care is defined as having two principal dimensions: access and effectiveness [12]. Access means that the users get the care they need. Effectiveness has two components: *effectiveness in clinical care*, which has a biomedical focus, and *effectiveness in interpersonal care*, which refers to interaction between health care professionals and users. The residential care staff have a central role in providing for the health care needs of people with PIMD, like management of pain, posture, nutrition, and ventilators, why their perspectives are unique and worth to study. Therefore, the aim of this study was to explore residential care staff and manager’s experiences and views of quality of health care services for adults with PIMD.

## Method

The design of the study is qualitative, but the study overall is exploratory.

### Participants

Residential care staff and managers were included in the study to acknowledge the perspectives of professionals who are essential care providers in adults with PIMD. The care staff are closest to the person with PIMD, providing care, whereas managers are responsible for the financial and personnel resources at the accommodation and thus have power over the care situation. The inclusion criterion for participation for both managers and care staff was that they had worked with people with PIMD for at least one year. To find eligible participants, residential care homes that provide services for adults with PIMD were identified by professionals at the adult habilitation services in a county in central Sweden. Residential care homes were selected based on geographical distribution creating a heterogeneous sample. The residential care homes were group homes with four to six adult residents with PIMD and 24-h support from care staff. The managers at these residential care homes were contacted and informed about the project; they were asked both to participate in the study and to help with the recruitment of care staff. The managers informed their care staff and asked them to contact the research group if they were willing to participate. The aim was to recruit equal numbers of residential care managers and members of the care staff. In total, 13 informants agreed to be interviewed: six managers and seven members of the care staff, see Table 1. Professionals from seven residential care homes covering rural (*n* = 6) and urban (*n* = 1) areas in Sweden were included in the study.

**Table 1:** Characteristics of the informants

	**Residential care managers ** *n* = 6	**Residential Care staff** *n* = 7
Age, mean (years) (range)	48.8 (35–62)	49.3 (32–66)
Sex, n		
Women	4	7
Men	2	0
Years in profession, (range)	9.3 (3–20)	17.7 (1–39)
Education level, n		
Upper secondary school < 12 years	0	7
Upper secondary school and further education^a^	3	0
University degree^b^	3	0

^a^≥ 13 years education

^b^Any degree from bachelor to doctorate

The Regional Ethical Review board in Uppsala approved this study (reference no. 2018/110). All informants signed a written informed consent to participate.

### Procedure

Semi-structured interviews were chosen and a topic guide was designed [13]. This guide covered six aspects of care: accessibility, treatment, cooperation, communication, support and participation. The interview guide was piloted with one residential care manager. That interview was not included in the study. An experienced physiotherapist conducted all interviews with an assistant, a registered nurse, present to ask follow-up questions and to seek clarification of the informants’ answers. Both had experience of working with adults with PIMD. The informants were invited to choose location for the interview, which was at their office, in the residential care facility, or at the adult habilitation services. The interviews lasted 60–90 min, were audio-recorded and transcribed verbatim by a secretary at the research centre. Data collection took place from October 2018 until January 2019. The interviewees were asked not to mention the adult with PIMD by name, because it was not relevant to the aim of this study and as a way to protect the person with PIMD’s anonymity. When the researchers judged that saturation had been reached, no further interviews were conducted [13].

### Analysis

An inductive qualitative content analysis was employed [14]. The inductive approach was chosen as it allows an open-minded and transparent exploration of research areas with little existing data [15]. The data were structured using the software program NVivo12 (QSR International, Inc., Cambridge, MA). The analysis was guided by Graneheim and Lundman [14], who described five analytic steps: meaning units, condensed meaning units, codes, categories and themes. In this study, we also used five steps but excluded condensed meaning units because many of the informants gave brief answers, even to follow-up questions, which meant that the meaning units were short from the beginning. Subthemes and one main theme were used instead of only themes. The first three steps including meaning units, codes and categories were related to the manifest content, while the last two steps, subtheme and theme, were made through interpretations in a latent level.

The whole analysis process was conducted by the first author, with intermittent discussions with the second author regarding adjustment of meaning units, codes and categories. When creating subthemes from the categories, and the theme both authors first worked individually and then discussed content until consensus was reached. This process was repeated with the subthemes to establish the theme. Finally, the results were verbally discussed between the authors, who did the analysis and the interviewers to ensure the validity of the analysis [16].

## Results

The theme that emerged from the analysis was ***Quality of health care is enhanced through residential care staff***. The care staff found themselves taking on a large responsibility to speak for the interests of the individual with PIMD in a complex care situation involving different organisations and professionals. An important aspect of the staff’s role is to enable as much participation as possible to the person with PIMD in decisions about care. Adults with PIMD often have few close friends and relatives around them who can be proxies in these decisions. Therefore, the staff often are essential. They know about the person with PIMDs communication skills and can use them in the best way.

Four subthemes emerged, and each subtheme consisted of several categories (Table 2).

**Table 2:** The categories, the subthemes and the theme

**Categories**	**Subthemes **	**Theme **
1. Ensure adaptation and responsiveness to the individual 2. Support is created in collaboration 3. Participation is enabled by advocacy	Individually tailored support promotes quality	Quality of health care is enhanced through residential care staff
1. Limitation by priorities and staffing 2. Need of support in acute situations 3. Flexibility and clarity are important in support services 4.Technical solutions can improve access to services	Accessibility requires adaptation and prioritization by healthcare providers	
1. Specific disability competence is essential 2. Continuous staff training important	Disability competence promotes quality and safety	
1. Collaboration is obstructed by laws and hierarchical structures 2. Access to a variety of professions enhance quality of care 3. Adult habilitation services are a resource for collaboration 4. Communication between residential care staff is crucial	Complex collaboration conditions between the person with PIMD, residential care staff and health care	

In the following sections, the categories and subthemes are described and quotes from informants are used to illustrate the categories.

### Individually tailored support promotes quality

According to the informants, individually tailored health care support is an important aspect of high-quality care for people with PIMD, which was described in three categories.

The informants pointed out that nurses, occupational therapists and physiotherapists make regular visits to the residential care facility to **ensure adaptation and responsiveness to the individual**. Because of their regular attendance they get to know the person with PIMD and his or her living conditions, which increases the chances of being responsive and creating solutions that suit the individual.

Almost every informant commented that, above all, the nurses from the municipality who are assigned to each residential care facility play a key role. They are the connection between the care staff and the healthcare system. Their importance became evident when a new nurse took over the role or when nurses with less experience were temporarily on duty, resulting in lower quality. These less experienced nurses had a harder time deciding how to handle the issues that arose and the residential care staff felt a greater responsibility.

*If there are nurses on site who recognise us, then it works well. then maybe it felt a bit like we had a lot of responsibility to tell them exactly, and this business about pneumonia and epilepsy and that some drugs are to be given only when needed. You [as residential care staff] have a very big responsibility to make it all work. (Informant 12 RCS)*

Individually tailored **support is created in collaboration with the individual**, built on knowledge about the particular disability and context of the adult with PIMD. This special knowledge about the person could help the care staff to achieve communication and hence quality of care. Care with an individually adapted perspective means respecting and affirming the person’s experience and interpretation of health and illness, as well as working to promote health, based on what health means to this particular person. In this case, there was a need for care staff to help interpret and elucidate the health experiences of the individual with PIMD and communicate this to the environment for example to the health care system. They thought their role was important and that other professionals underestimated its significance when considering who should have the responsibility to represent the person with PIMD in health care issues. Moreover, they thought there was no one else who could represent these individuals with the same insight.

Coordinated individual plans were described as a practical tool to individualize care. Individual plans are set in a meeting with staff involved with the person with PIMD, who is also present, to express and decide what needs they should work together to fulfil. The focus is on the individual in order to plan the best support for him or her in the long run. The residential care staff felt like they had a special role because they know the person with PIMD best of all that participated in the meeting.

*Well I’ve been in some [coordinated individual plan meetings] and I’ve been in some networking meetings around people [with PIMD], concerning both daily activities and residential care. And where I think we have good discussions and that you are open and that, that the people at the habilitation service don’t have a monopoly on the truth but they actually listen to us and try to be curious and get to know the person [with PIMD]. Because we know our residents very, very well, you know. (Informant 9 RCM)*

The third category, **participation is enabled by advocacy**, signifies that both the care staff and the managers felt that their primary mission was to represent the individual with PIMD in meetings they attended together with the individual. Their primary task was to represent the individual in order to make wise care decisions and to help them to be involved in the discussion. Their experiences indicated that their advocacy made it possible for the individual with PIMD to participate.

This was experienced as a great responsibility that the care staff took and that no one else could manage. They described the challenge of not talking over the individual’s head. They also described difficulties in interpreting the person’s gestures and movements and then conveying the meaning to the others present. If they made the wrong interpretation, the whole situation for the individual with PIMD could develop into something that the person did not want.

*After all, it’s so easy to talk over their heads. And in fact, this can be the most difficult job for the staff, these very people who do not communicate through speech and perhaps very little gestures and movements and sounds, and constantly trying to tick off what they want, is this good or can it be better. /…/ Also, that the staff who work closest have a huge responsibility to interpret and convey what they notice, feel and see to that we, the people around, can work to get them as involved as possible. (Informant 8 RCM)*

### Accessibility requires adaptation and prioritization by healthcare providers

This subtheme reflects the value that care staff and managers place on having a flexible and adaptive approach towards the person with PIMD within different areas such as treatment, costs and technology to achieve quality of care. Four categories form this subtheme.

Access to care is limited for persons with PIMD is **limited by priorities and staffing**. The informants pointed out that adequate care for individuals with PIMD requires a lot of staff, and the staffing resources set the limits for what can be done for the individual; this meant that these resources had to be prioritised. However, the informants were not of the opinion that more staff would be guaranteed to solve any particular problem, but rather that, in general, having more resources helps them to give more adapted care.

The informants experienced a **need of support in acute situations**. Typically, simpler health issues were handled by the residential staff because of difficulties getting access to health services. When in acute need of support from external services, there was often a need to be persistent and keep on seeking services to ensure that the individual got adequate help.

*We have experienced that one of our residents got blisters all over his body and we didn’t get any special help with it. But then I sat at the health clinic until we got help. Informant 5 RCS)*

Another aspect of accessibility was that **flexibility and clarity are important in support services**. Some of the informants described the adult habilitation service as an important collaborator, but experiences differed concerning the accessibility of those services. An important issue for accessibility was the aspect of meeting habilitation professionals on equal terms without any authoritarian attitudes affecting the communication. If equal terms could be established between the care staff, the staff from the adult habilitation service and the individual with PIMD, accessibility was described as successful.

*So in general it works well /… / but there is probably the time aspect /…/ and that there are many who want help from the adult habilitation service so that even if we can see that our residents would need this /… / have to miss out and there are queues and so on. (Informant 1 RCM)*

Several informants observed that **technical solutions can improve access to the support service**. Sometimes it was important to easily and quickly get in touch with professionals in the health care services. This could be enhanced by technical solutions such as an e-mail address or a telephone number where you can reach an entire unit.

### Disability competence promotes quality and safety

This subtheme emphasises the importance of the right education and training for quality of care. The care staff have to develop their competence to meet every individual’s unique needs and desires. Two categories form this subtheme.

To ensure **specific disability competence is essential** which means knowledge about the disability and its consequences for the individual. Informants reported that they encounter a lot of ignorance and prejudice about people with different types of disabilities. Knowledge makes it easier to get individuals with PIMD involved and provide the support they need.

*I think that it’s a bit like this, in fact, that in many professions, perhaps they haven’t talked much about people with disabilities in their training. Because I think when, I myself have worked in hospitals once upon a time and worked at the children’s clinic then, and I noticed quite quickly that there were people who were very uncomfortable when people with disabilities were admitted to the ward. /…/ and they didn’t really know how to deal with these people either. (Informant 6 RCM)*

One of the informants described feeling as if she were leaving a small vulnerable child when an adult resident with PIMD had to be admitted to hospital. She argued that it is important that the care staff with competence about the individual with PIMD should be allowed to support the individual in hospital. This could increase the likelihood of the person with PIMD participating in a care decision. One manager observed that middle-aged people with PIMD are used to other people making decisions over their head. They hadn’t been invited to participate or been asked their opinion during their childhood.

Living with PIMD is complex, and the **staff members need continuous training**. The care staff have many issues to keep in mind. They also have to repeat instructions often for new members of staff and when new instructions are received from the habilitation centre or from the nurses in the municipality, for example.

*There I think my staff is very good at signalling if they have not understood what it is exactly they should do or how this should work or suchlike. So then they are pretty good at contacting the habilitation services. (Informant 9 RCM)*

### Complex collaboration conditions between the person with PIMD, the residential care staff and the health care

To ensure good quality of care for individuals with PIMD, collaboration between different services is almost always required, as well as collaboration between different professions, which together create a complex situation. This subtheme consists of four categories.

In the category **collaboration is obstructed by laws and hierarchical structures**, the informants explained that the Secrecy Act can sometimes hinder care staff from speaking with other professionals, for example in health care, about individuals with PIMD because they work in different organisations. There are also practical obstacles, such as finding it hard to make time for meetings, and disagreements about which health care service should perform what and who is responsible.

*It can sort of be difficult to practically synchronise times to meet. And that there are waiting times in some services and we perhaps more want it to happen as soon as possible. And sometimes there are boundaries too, so who does what and who is responsible for what, that might be a little fuzzy. (Informant 8 RCM)*

Some of the informants felt that the hierarchical structures often had a negative impact on this collaboration. They emphasized that the care staff should be persistent, stand on the individual’s side and express opinions to ensure the individual’s needs were met.

An idea that arose was that close **access to a larger variety of professions would enhance quality of care** if there were multi-professional teams connected to the residential care facility. Then a better overall view of everyday life, a continuity, could be created around the individuals who need the support.

*And then if something happens, well then they bring in someone else or someone who isn’t part [of the team] in the same way because it is not relevant. Sometimes you would wish that you had the same team around everyone who lived in the same group home. It isn’t like that for us. (Informant 2 RCM)*

**The adult habilitation services are a resource for collaboration**, because they have education, competence and resources for creating cooperation between services. They have extensive experience of leading planning meetings for care with people with PIMD. Together with other services, the care staff and the people with PIMD they could improve the division of labour in the habilitation activities.

The informants felt that **communication among residential care staff is crucial for quality of care** They described several issues concerning communication and information, which could be within the group of care staff, with other health care organisations or with others who need information about the individual with PIMD. It was easy to forget to pass on important information to staff members who worked different shifts. Information was considered an important prerequisite for good quality of care; however, it was considered a challenge to ensure a satisfying communication.

*No, this communication business is very difficult, it is almost the hardest thing we do in my opinion. That the right information goes out and that everyone gets it /… / and what we do is that we raise it at our staff meetings at our group homes then. (Informant 9 RCM)*

Finally, some informants expressed surprise and wonder that the challenging communication situation around the individuals with PIMD works as well as it does. They noted that the professionals in other services are often good at dealing with individuals with PIMD even if they seldom meet them.

## Discussion

This study explored the experiences of residential care staff and managers concerning quality of care in health care services for adults with PIMD. The results showed that the residential staff feel themselves to be essential to ensure quality of care in a complex care situation, requiring sufficient competence to allow adaptation to the individual with PIMD; flexibility on the part of the health care providers was essential to facilitate this quality of care.

Our finding that the care staff are the most important factor for ensuring quality of care might not be surprising given that the informants were the care staff themselves and their managers, and because of this, the validity of that result might be found questionable. Nevertheless, people with PIMD have substantial support needs for communicating even their most basic wishes, which leaves them in a precarious situation where only those who are closest to them, are able to interpret and understand them. Our study illustrates that residential care staff thereby become the representatives for people with PIMD to enable access to services, their participation in the health care process and quality of care. That professionals have the ability to give meaning to participation in these adults is supported by a study operationalizing the concept of participation [17] for adults with visual and severe or profound intellectual disabilities in support person’s experience [18, 19].

Considering the important role that the care staff have, in their own view, with great responsibility for another person’s health, well-being and participation in society, they think of their role as underestimated. A position as a residential care worker does not require a long training or educational qualifications. Based on our results, we propose that continued on-the-job training for care staff, covering disabilities and the types of services they require, might lead to improvement of the quality of care. People with PIMD are among the most marginalised groups in society [20], which is reflected in the low status of the work of the care staff and their correspondingly low income.

One tool to handle the complex situation that our informants emphasised strongly was how important individual support planning (ISP) meetings were for the people with PIMD and the staff around them. An ISP meeting helps the staff and the individual with PIMD to follow a protocol and it results in a document containing decisions [21]. The ISP also contains goals for the person with PIMD [22]. In these meetings, all the involved staff and often the person with PIMD participate. The informants in our study also reported that the ISP contributes to better collaboration between professions and services.

Professionals who provide health care to people with PIMD have to work in close collaboration with the care staff and the person with PIMD to build a relationship and a familiarity with the person’s health care needs. They have to know the person with PIMD and the environment they live in, developing an understanding of the work situation of the care staff, to enable a good quality of care. Similar results were found in another study, illuminating the importance of building a relationship of respect for the people with PIMD and the care staff who can represent them [23]. Our results suggest that if the health care providers take the time and make the effort to understand this complex situation, the quality of care might increase. This close relationship between care staff and persons with PIMD is also important to build to enhance participation for the target group. Participation was shown in another study to be dependent on several conditions [24]. The experience by staff members and managers was dependent upon the conditions belonging to the adult with PIMD, the understanding of the managers and staff and the organisation to achieve participation. This is also findings from our study.

Several of the informants in the present study described their experiences of the hierarchical structure, in which the care staff who represent the adults with PIMD perceive that they are being treated as less important than the professionals in the health care system. In a study with patients and nurses in a hospital setting, a similar power relation was described [25]. That study showed that patients expressed various experiences, but their voices were not taken into account and the hospital system instead listened to health care provider expertise. Griscti et al. [25] further found that the hierarchical structure also encouraged nurses to become gatekeepers of the service who excluded patients from access to care. The health care system needs to be observant of how these hierarchical structures position their professionals in a power relationship to their patients and the patients´ representatives.

One central group of health professionals described in our study are the municipality nurses who function as a link between care staff and health care services. Their role is crucial because they have more medical competence and responsibility than the care staff, which enhances quality of care and ensures medical safety. The nurses are also responsible for the documentation that is required for medical issues. Van der Heide et al. [26] argued that documentation is essential to ensure safety and could compensate for the communication problems. Therefore, close contact with the municipality nurse seems to be important both for the quality of care and medical safety of people with PIMD. The need to educate nurses about how to communicate with people with PIMD has previously been identified [27], which further highlights the unique knowledge the care staff have about their service users. Fitzgerald & Sweeney [28] suggested specialist training to address the complex interplay that these nurses needed to master in their work situation. We also suggest that other professions working with care staff and people with PIMD need similar training.

### Methodological considerations

The exploratory character of the study can be addressed as a limitation including the small sample size. It is conceivable that the results can be applied to other disability groups who have difficulty drawing attention to their own case and need the support of others for this. Another issue to consider is whether the experiences and results are transferable to other groups of care staff, professionals and relatives of people with PIMD; further studies to investigate this are planned.

The interviewers in this study were employees of the adult habilitation services within the region. Interviewers did not interview managers or staff to whom they had a professional relationship, but still, it cannot be ruled out that informants withheld criticism of the habilitation services due to the interviewers’ employment. On the other hand, these two interviewers had extensive experience of habilitation and of individuals with PIMD so they could ask relevant follow-up questions, which likely contributed to richer interview material.

### Further research

This study is, to our knowledge, the first to explore quality of health care services for people with PIMD, focusing on the experience of care staff and their managers. The topic of quality of care for people with PIMD needs to be further explored from other perspectives, such as their personal assistants and close relatives.

## Conclusion

This study shows that the residential care staff are essential to ensure quality of care in different types of health care for people with PIMD. They work in complex collaboration networks and they have to be flexible and adaptive. They develop unique competence about the individual with PIMD and the disability, enabling them to tailor care solutions to each individual and thus create quality of care. But more knowledge is needed about how to ensure quality of care for people with PIMD from different perspectives, for example regarding the experiences of personal assistants and close relatives of people with PIMD.

## Data Availability

The interviewed guide used during the current study is available from the corresponding author on request. The datasets generated and/or analyzed during the current study are not publicly available due to the risk of participants being identified in interview material but are available from the corresponding author on reasonable request.

## References

[1] Kamstra A, Van der Putten A, Maes B, Vlaskamp C (2019). Exploring spontaneous interactions between people with profound intellectual and multiple disabilities and their peers. J Intellect Dev Disabil.

[2] Nakken H, Vlaskamp C (2007). A need for a taxonomy for profound intellectual and multiple disabilities. J Policy Pract Intellect Disabil.

[3] Ölund A (2012). Medicinsk omvårdnad vid svåra flerfunktionshinder: handbok [Medical Nursing in profound intellectual and multiple disability: Manual].

[4] Van Timmeren EA, Van der Putten AAJ, Van Schrojenstein Lantman-de Valk HMJ, Van der Schans CP, Waninge A (2016). Prevalence of reported physical health problems in people with severe or profound intellectual and motor disabilities: A cross-sectional study of medical records and care plans. J Intellect Disabil Res..

[5] Van Timmeren EA, Waninge A, Van Schrojenstein Lantman-de HMJ, Van der Putten AAJ, Van der Schans CP (2017). Patterns of multimorbidity in people with severe or profound intellectual and motor disabilities. Res Dev Disabil.

[6] Borgström E, Carlberg AC (2010). Till mångas nytta. Om behovet av ett nationellt kunskapscenter för frågor om flera och omfattande funktionsnedsättningar [To the benefit of many. About the need for a national knowledge center for questions about several and extensive disabilities].

[7] Petry K, Maes B, Vlaskamp C (2001). Developing a procedure for evaluating quality of life for people with profound and multiple disabilities. Tizard Learning Disability Review.

[8] Maes B, Lambrechts G, Hostyn I, Petry K (2007). Quality-enhancing interventions for people with profound intellectual and multiple disabilities: a review of the empirical research literature. J Intellect Dev Disabil.

[9] Vlaskamp C, Fonteine H, Tadema A, Munde V (2009). Manual for the "Alertness in people with profound intellectual and multiple disabilities" checklist.

[10] Petry K, Maes B, Vlaskamp C (2007). Support characteristics associated with the quality of life of people with profound intellectual and multiple disabilities: The perspective of parents and direct support staff. J Policy Pract Intellect Disabil.

[11] Beadle-Brown J, Leigh J, Whelton B, Richardson L, Beecham J, Baumker T (2016). Quality of life and quality of support for people with severe intellectual disability and complex needs. J Appl Res Intellect Disabil.

[12] Campbell SM, Roland MO, Buetow SA (2000). Defining quality of care. Soc Sci Med.

[13] Kvale S, Brinkmann S. InterViews: Learning the craft of qualitative research interviewing. 2, editor. Los Angeles Sage Publications; 2009.

[14] Graneheim UH, Lundman B (2004). Qualitative content analysis in nursing research: Concepts, procedures and measures to achieve trustworthiness. Nurse Educ Today.

[15] Elo S, Kyngäs H (2008). The qualitative content analysis process. J Adv Nurs.

[16] Hsieh H-F, Shannon SE (2005). Three approaches to qualitative content analysis. Qual Health Res.

[17] Granlund M, Imms C, King G, Andersson A, Augustine L, Brooks R (2021). Definitions and operationalization of mental health problems, wellbeing and participation constructs in children with NDD: distinctions and clarifications. Int J Environ Res Public Health.

[18] Hanzen G, Van Nispen RM, Van der Putten AA, Waninge A (2017). Participation of adults with visual and severe or profound intellectual disabilities: Definition and operationalization. Res Dev Disabil.

[19] Hanzen G, Waninge A, Vlaskamp C, Van Nispen RMA, Van der Putten AAJ (2018). Participation of adults with visual and severe or profound intellectual disabilities: analysis of individual support plans. Res Dev Disabil.

[20] Gittins D, Rose N (2008). An audit of adults with profound and multiple learning disabilities within a West Midlands Community Health Trust – implications for service development. Br J Learn Disabil.

[21] Matousova-Done S, Gates B, Gates B (2006). The nature of care planning and delivery in intellectual disability nursing. Care planning and delivery in intellectual disability.

[22] Herps MA, Buntinx WHE, Schalock RL, van Breukelen GJP, Curfs LMG (2016). Individual support plans of people with intellectual disabilities in residential services: content analysis of goals and resources in relation to client characteristics. J Intellect Disabil Res.

[23] Vlaskamp C, De Geeter KI, Huijsmans LM, Smit IH (2003). Passive activities: the effectiveness of multisensory environments on the level of activity of individuals with profound multiple disabilities. J Appl Res Intellect Disabil.

[24] Talman L, Wilder J, Stier J, Gustafsson C (2019). Staff members and managers' views of the conditions for the participation of adults with profound intellectual and multiple disabilities. J Appl Res Intellect Disabil.

[25] Griscti O, Aston M, Warner G, Martin-Misener R, McLeod D (2017). Power and resistance within the hospital's hierarchical system: the experiences of chronically ill patients. J Clin Nurs.

[26] Van der Heide DC, Van der Putten AA, Van den Berg PB, Taxis K, Vlaskamp C (2009). The documentation of health problems in relation to prescribed medication in people with profound intellectual and multiple disabilities. J Intellect Disabil Res.

[27] Healy D, Walsh PN (2007). Communication among nurses and adults with severe and profound intellectual disabilities: predicted and observed strategies. J Intellect Disabil.

[28] Fitzgerald MD, Sweeney J (2013). Care of adults with profound intellectual and multiple disabilities. Learn Disabil Pract.

[29] Declaration of Helsinki – Ethical Principles for Medical Research Involving Human Subjects, (2017). 19886379

